# IR spectroscopic characterization of 3d transition metal carbene cations, FeCH_2_^+^ and CoCH_2_^+^: periodic trends and a challenge for DFT approaches†

**DOI:** 10.1039/d4cp00026a

**Published:** 2024-02-07

**Authors:** Frank J. Wensink, Corry E. Smink, P. B. Armentrout, Joost M. Bakker

**Affiliations:** a Institute for Molecules and Materials, FELIX Laboratory, Radboud University Toernooiveld 7 6525 ED Nijmegen The Netherlands joost.bakker@ru.nl; b Department of Chemistry, University of Utah 315 South 1400 East Salt Lake City Utah 84112 USA armentrout@chem.utah.edu

## Abstract

A combination of IR multiple-photon dissociation (IRMPD) action spectroscopy and quantum chemical calculations was employed to investigate the [M,C,2H]^+^ (M = Fe and Co) species. These were formed by reacting laser ablated M^+^ ions with oxirane (ethylene oxide, c-C_2_H_4_O) in a room temperature ion trap. IRMPD spectra for the Fe and Co species are very similar and exhibit one major band. Comparison with density functional theory (DFT) and coupled cluster with single and double excitations (CCSD) calculations allows assignment of the spectra to MCH_2_^+^ carbene structures. For these 3d transition metal systems, experimental IRMPD spectra compare relatively poorly with DFT calculated IR spectra, but CCSD calculated spectra are a much better match primarily because the M–C stretch gains significant intensity. The origins of this behavior are explored in some detail. The present results are also compared to previous results for the 4d and 5d congeners and the periodic trends in these structures are evaluated.

## Abstract 2

A combination of IR multiple-photon dissociation (IRMPD) action spectroscopy and quantum chemical calculations was employed to investigate the [M,C,2H]^+^ (M = Fe and Co) species.

## Graphical abstract

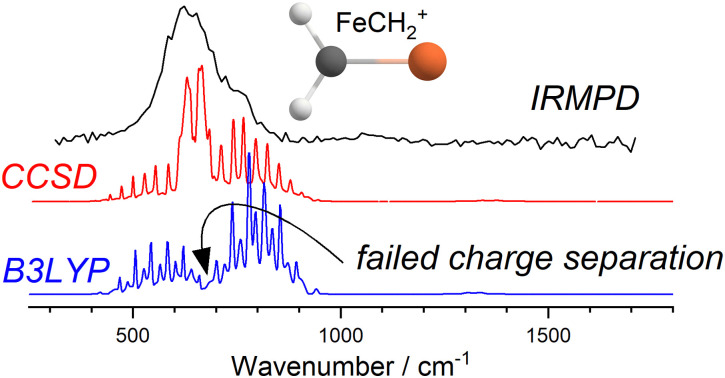

## Introduction

Research investigating the functionalization of the C–H bonds of alkanes has a long history.^1^ The bottleneck in making such a process both energy-efficient and selective is the activation of the strong C–H bonds. Methane is the most commonly studied alkane, largely because it is a major component of natural gas and the simplest alkane molecule. Selective C–H bond activation is particularly important because it is desirable to convert methane into liquid fuels like methanol that remain energy rich but are more easily transported. In general, the direct use of methane in catalytic processes is not presently pursued because the available processes are not sufficiently specific or energy efficient. Instead, the starting point is often thermal cracking, after which the available products are used to synthesize more valuable chemicals. The direct utilization of methane still requires the development of more effective C–H bond activation catalysts, especially those for earth abundant metals.

Potentially, studies of models systems of active catalyst sites provide a means to rationally design the needed catalysts. Model systems can provide a more detailed description of the fundamental interactions between methane and the active site,^2^ allowing insight into how to manipulate the site for better efficiency and/or selectivity. Isolation of such model systems in the gas phase allows the most detailed information on the intrinsic interaction to be obtained as the perturbing effects of solvent or support are absent. Previous studies have found that several third-row (5d) transition metal (TM) cations, Ta^+^, W^+^, Os^+^, Ir^+^, and Pt^+^, will activate methane at room temperature. These dehydrogenation reactions lead to formation of [M,C,2H]^+^ products.^3–7^ IR spectroscopic characterization of these [M,C,2H]^+^ products showed that [Ta,C,2H]^+^ and [W,C,2H]^+^ have MCH_2_^+^ carbene structures but are unsymmetrical because of distortions induced by agostic interactions.^8–10^ In contrast, [Pt,C,2H]^+^ has a carbene structure with *C*_2v_ symmetry. Although Ir^+^ also exhibited experimental evidence for a symmetric IrCH_2_^+^ carbene structure, the main species formed for both [Os,C,2H]^+^ and [Ir,C,2H]^+^ was a hydrido metal carbyne HMCH^+^.^8–11^ Notably, the favored HIrCH^+^ structure had been discarded on the basis of theoretical relative energies,^12^ demonstrating the need for experimental spectroscopic characterization. In contrast to third-row TM cations, none of the first-row (3d) TM cations dehydrogenates methane exothermically,^5,13–16^ and although the second-row (4d) TM cations, Zr^+^ and Nb^+^ have been reported to dehydrogenate methane at room temperature,^5,17^ guided ion beam studies demonstrate that such reactions are endothermic for all 4d TM cations.^15,18,19^ To understand the underlying reasons why the reactions of the lighter 3d TM cations with methane are unproductive and endothermic, it is of interest to characterize the structures of [M,C,2H]^+^ products where M is a first-row TM.

Because Os^+^ and Ir^+^ were shown to be the most reactive 5d TM ions for methane dehydrogenation and also show the most diversity in their structures,^4,6–9,11^ we have previously studied the bonding nature and structures of [M,C,2H]^+^ with their second-row congeners: the elements of groups 8 and 9, M = Ru and Rh.^20^ The present study continues this examination of periodic trends by focusing on the first-row congeners, M = Fe and Co. Earlier studies investigated the M^+^–CH_2_ bond strengths for first-row TMs and found that the bond dissociation energy (BDE, *D*_0_) decreased with an increase in the promotion energy (required for promoting a d electron to an s orbital) of the atomic metal cation.^21^ The iron carbene cation BDE, *D*_0_(Fe^+^–CH_2_), was determined to be 3.53 ± 0.04 eV in guided ion beam tandem mass spectrometry (GIBMS) experiments^22,23^ and ≤3.54 ± 0.02 eV by photodissociation,^24^ such that formation of FeCH_2_^+^ + H_2_ from reaction with methane is endothermic by 1.21 ± 0.04 eV given *D*_0_(CH_2_–H_2_) = 4.743 ± 0.001 eV.^25^*D*_0_(Co^+^–CH_2_) was experimentally determined to be 3.29 ± 0.05 eV by GIBMS^16^ and ≤3.43 ± 0.02 eV by photodissociation,^24^ such that dehydrogenation of methane requires 1.46 ± 0.05 (≥1.31 ± 0.02) eV. In both the iron and cobalt systems, the cross sections measured for the M^+^ + CH_4_ → [M,C,2H]^+^ + H_2_ reactions had threshold energies that exceeded the endothermicities, clearly showing the presence of an energy barrier, which is more than likely found in the exit channel.^16,23^

Elsewhere, CCSD calculations on the [M,C,2H]^+^ species indicate that FeCH_2_^+^ has ^4^B_1_ and ^4^B_2_ electronic states that are nearly degenerate, similar to CoCH_2_^+^, which has nearly degenerate ^3^A_1_ and ^3^A_2_ electronic states.^26^ A potential energy surface (PES) for reaction between Co^+^ and CH_4_ identified the physisorbed Co(CH_4_)^+^ complex as the only intermediate that could be formed exothermically along the surface leading toward CoCH_2_^+^ + H_2_.^16,27^ A similar conclusion holds for the reaction between Fe^+^ and CH_4_.^23,28^

Because neither Fe^+^ nor Co^+^ reacts exothermally with methane,^14,16^ we react the corresponding M^+^ ions with oxirane (ethylene oxide, c-C_2_H_4_O) in order to form the [M,C,2H]^+^ of interest. Oxirane readily reacts with many TM cations because of its ring strain and because [M,C,2H]^+^ formation is accompanied by the stable CH_2_O neutral product. As a consequence, extraction of CH_2_ from oxirane requires much less energy than from methane, *D*_0_(CH_2_–CH_2_O) = 3.375 ± 0.004 eV, compared to *D*_0_(CH_2_–H_2_) = 4.743 ± 0.001 eV.^25^ Thus, the thermochemistry noted above indicates that formation of MCH_2_^+^ + CH_2_O in the reaction of M^+^ with oxirane, is exothermic by 0.16 ± 0.04 eV for M = Fe. For M = Co, the reaction is either slightly endothermic by 0.08 ± 0.05 eV or exothermic by ≤0.06 ± 0.02 eV. Indeed, the reaction of Fe^+^ with oxirane has been shown to result in formation of both FeO^+^ and FeCH_2_^+^ at thermal energies,^29^ whereas Co^+^ + oxirane results mainly in CoCH_2_^+^, CoCO^+^, and CoC_2_H_4_O^+^.^30^ In that study, the kinetic energy dependence of the CoCH_2_^+^ cross section was consistent with a slightly endothermic process but the reaction is still appreciable (reaction efficiency near 10% at thermal energies).

In the present work, we probe the structure of the [M,C,2H]^+^ product ions for M = Fe and Co using a combination of infrared multiple photon dissociation (IRMPD) action spectroscopy and theoretical calculations. IRMPD spectra are recorded using the same Fourier transform ion cyclotron resonance (FTICR) mass spectrometer recently used to show that the reaction of Pt^+^ with two methane molecules leads to the formation of a Pt^+^(ethene) complex following two dehydrogenations and C–C coupling.^31^ In that same work, we demonstrated that the spectrum of [Pt,C,2H]^+^ recorded with this instrument is consistent with that reported previously,^8^ where [Pt,C,2H]^+^ was formed in a molecular beam environment without mass-isolation prior to irradiation. Earlier attempts to record IRMPD spectra of [M,C,2H]^+^ with M = Fe and Co were unsuccessful in the molecular beam apparatus because the ion intensities were insufficient. Here, we take advantage of the possibility to react TM^+^ with c-C_2_H_4_O over longer times and to mass-isolate the formed [M,C,2H]^+^ species in order to obtain the desired spectra.

Critically, these small ions also form benchmark systems for the accuracy of the quantum chemical methods. Previously, we found that relatively standard density functional theory (DFT) methods proved sufficient to accurately predict the IR fingerprints, both in terms of frequencies and IR intensities, necessary for assigning the experimental IR spectra of [M,C,2H]^+^ species with M 5d elements.^8–11,31^ For the 4d elements M = Ru and Rh, the same conclusion holds, even though the assignment for the [Rh,C,2H]^+^ species was complicated by the multi-reference character of RhCH_2_^+^.^20^ In contrast, here, we find that these comparisons are not so straightforward for 3d elements. We therefore employ higher-level methods to interpret the observed spectra.

## Methods

### Experimental

The experimental procedure here mirrors that followed in our recent work on the similar [Ru,C,2H]^+^ and [Rh,C,2H]^+^ species.^20^ The atomic metal (Fe, Co) cations were formed by irradiating a solid metal target disk with a frequency doubled Nd:YAG laser at 30 Hz.^32^ Helium gas was injected by a pulsed valve in order to collisionally cool the metal ions and guide them to an orifice where the gas mixture adiabatically expanded into vacuum, further cooling all species. The ions were transferred by a radio-frequency (rf) only quadrupole mass filter, to a rf quadrupole ion trap having rectangular electrodes. There the ions were trapped by collisions with 5 × 10^−4^ mbar of argon buffer gas, which contained a partial pressure of 1 × 10^−6^ mbar of oxirane. After accumulating for approximately 400 ms, atomic metal cations and product ions were released from the trap by lowering the voltage on the exit electrode of the ion trap. The ions were then guided into one of four cells of the FTICR mass spectrometer integrated with the cavity of the Free Electron Laser for IntraCavity Experiments (FELICE). The first FTICR cell (cell 1) was located in the laser focus and the fourth was 30 cm from the focus, which reduces the photon fluence by a factor of 14. After ion capture, unwanted masses were ejected from the FTICR cell using a combination of chirped and single-frequency rf excitation pulses,^33^ leaving only [M,C,2H]^+^ ions. Resonant absorption of IR photons at a vibrational band of the ion increased the internal energy of the ions leading to fragmentation. IRMPD spectra were recorded by monitoring intensities of precursor (*I*_p_) and fragment (*I*_frag_) ions present in the FTICR cell after irradiation. The resulting spectrum is provided as the fragmentation yield *Y*:
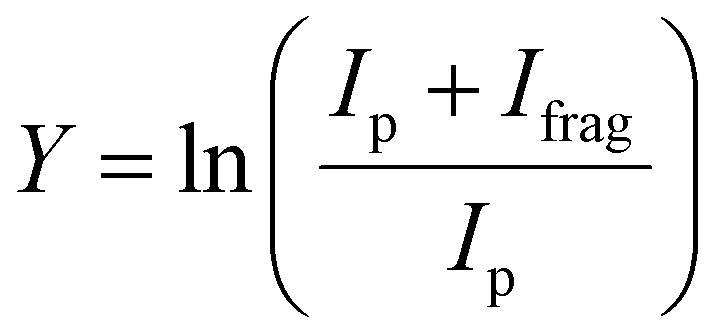


The IR light of FELICE was scanned over the 300–2000 cm^−1^ spectral range for the present work. FELICE produced micropulses at 1 GHz, with macropulses that lasted between 6–10 µs at a repetition rate of either 5 or 10 Hz. A single macropulse with the ions in FTICR cell 4 (low fluence) was used to record all spectra unless indicated otherwise. Macropulse energies of FELICE ranged from 0.3 to 0.5 J moving from low to high frequencies. This corresponds to macropulse fluences ranging from 0.9 to 6.6 J cm^−2^ in FTICR cell 4. The spectral bandwidth was near transform limited and the full-width at half-maximum (FWHM) was approximately 0.7% of the central frequency during the present experiments. To locate the band frequencies listed below, Gaussian curves were fitted to the experimental IRMPD spectra.

### Computational

All calculations were performed here using the Gaussian 16 software package.^34^ Generally, molecular structures for all species were located using unrestricted B3LYP hybrid functional and the def2-TZVPPD basis set, which treats all electrons explicitly for H, C, Fe, and Co.^35–37^ This combination was used because it has performed well in calculating the IR spectra of the 5d [M,C,2H]^+^ species and the M^+^–CH_2_ BDEs calculated at this level match experimental values relatively well: *D*_0_(Fe^+^–CH_2_) was calculated to be 3.66 eV (experimentally 3.53 ± 0.04 eV) and *D*_0_(Co^+^–CH_2_) = 3.24 eV (3.29 ± 0.05 eV), as outlined in Table 1. These calculated values (as well as all others provided below) include zero-point vibrational energies. True minima in the structures were verified by ensuring that the calculated harmonic vibrational frequencies were all real. For comparison with the experimental spectra, the unscaled theoretical harmonic vibrational frequencies had simulated rovibrational envelopes included by assuming pure a-, b-, or c-type transitions.^38^ To mimic the FELICE spectral bandwidth, the resulting rovibrational transition lines were convolved with a Gaussian line shape having a FWHM of 0.9% of the central frequency. To simulate additional broadening effects, convolutions were also done employing a 9% FWHM bandwidth.

**Table 1:** Fragmentation channels and dissociation energies (*D*_0_) of the lowest energy [M,C,2H]^+^ species

Species	Fragments	Bond dissociation energy *D*_0_ (eV)	
		Theory^a^	Experiment
FeCH_2_^+^	FeC^+^ + H_2_	3.30	2.86 ± 0.22^b^
	Fe^+^ + CH_2_	3.66	3.56 ± 0.22,^b^ 3.53 ± 0.04,^c^ ≤3.54 ± 0.02^d^
CoCH_2_^+^	CoC^+^ + H_2_	3.77	3.08 ± 0.22,^b^ 3.01 ± 0.30,^c^^e^^f^ ≤3.15 ± 0.30^d^^e^^f^
	Co^+^ + CH_2_	3.24	3.64 ± 0.22,^b^ 3.29 ± 0.05,^c^ ≤3.43 ± 0.02^d^

a Theoretical values are calculated at the uB3LYP/def2-TZVPPD level of theory.

b Ref. 39.

c Ref. 22.

d Ref. 24.

e Ref. 16.

f Uses *D*_0_(C–H_2_) = 3.320 ± 0.001 eV.^25^

Additional geometry and frequency calculations were done at second-order Møller–Plesset, MP2(full),^40^ and coupled cluster with single and double excitations, CCSD(full),^41^ levels (where full indicates correlation of all explicit electrons) using the same def2-TZVPPD basis set. These were followed by single point calculations with added perturbative triple excitations, *i.e.*, at the CCSD(T,full) level. For FeCH_2_^+^, calculations using several additional DFT approaches were also pursued.

Notably, no scaling factors were used to compare the theoretical spectra with experiment, which is unusual for these types of systems.^8–11,20,31,42^ Nevertheless, such scaling factors were not found to improve the agreement between theory and experiment and therefore are not used throughout this work.

## Results & discussion

Reactions between M^+^ (M = Fe, Co) and oxirane resulted in the product mass distributions shown in Fig. S1 of the ESI.† We observed ionic products [M,C,2H]^+^, MO^+^, [M,O,H]^+^, and [M,O,2H]^+^ for both M. For Fe^+^, the former two products were observed in earlier experiments,^29^ but the latter two were not. We assume these species are the result of reaction with a water contaminant present in the c-C_2_H_4_O gas or tubing. In the reaction between Co^+^ and oxirane, we do observe [Co,C,2H]^+^ and CoO^+^ but not the CoCO^+^ species observed before in a single bimolecular encounter.^30^ We also observe a [Co,C,3H]^+^ species (which would not have been observed easily in the low mass resolution ion beam experiments). Formation of CoCH_3_^+^ + HCO from Co^+^ + c-C_2_H_4_O is endothermic by 0.29 ± 0.04 eV.^22^ The absence or presence of these species might be a result of multiple reactions with c-C_2_H_4_O or involve electronic excitation of Co^+^. Observed [Co,O,2H]^+^ and [Co,O,H]^+^ species again are likely to result from reactions with a water contaminant.

### IR spectroscopy of FeCH_2_^+^

**Fig. 1:**
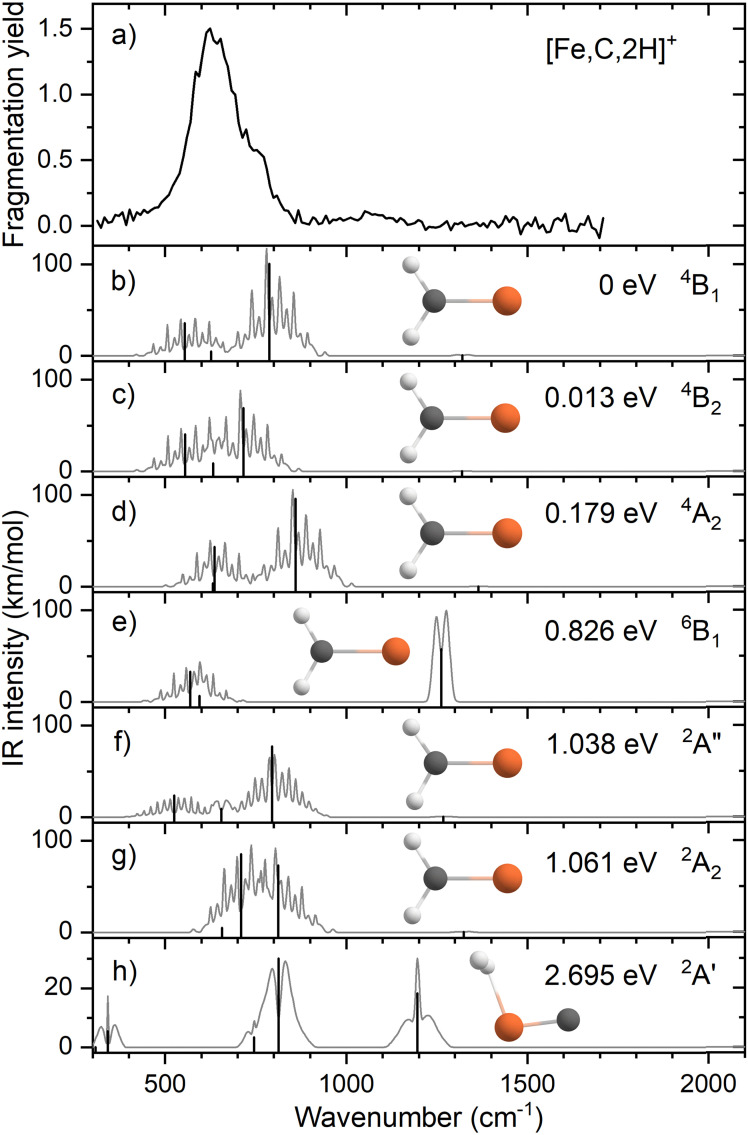
(a) Experimental IRMPD spectrum of [Fe,C,2H]^+^; (b)–(h) B3LYP/def2-TZVPPD calculated IR spectra of different [Fe,C,2H]^+^ states and isomers with the harmonic vibrations in black and the rovibrational envelopes in grey accompanied by geometric structure, relative energy, and electronic state.

Fig. 1a shows the experimental IRMPD spectrum of [Fe,C,2H]^+^ (*m*/*z* = 70) constructed using the ^56^Fe isotope of the two observed fragment masses Fe^+^ and FeC^+^. The spectrum is dominated by a strong and broad band peaking at 632 cm^−1^. This band has a shoulder on the high frequency side with a maximum near 767 cm^−1^. Fig. S2a of the ESI† shows a potential band could be discerned in only the Fe^+^ fragmentation channel just above 1000 cm^−1^. A careful analysis of the mass-spectra underlying the IR spectra shows that this band results from formation of Fe^+^ by fragmentation of a [Fe,C,3H]^+^ minor product that was not mass-eliminated before irradiation, see a typical example in Fig. S3 in the ESI.† As [Fe,C,3H]^+^ does not dissociate by loss of H_2_ + H, this band does not appear in the FeC^+^ fragmentation product spectrum. A second band for [Fe,C,3H]^+^ is observed to coincide with the main band of [Fe,C,2H]^+^, and therefore has no influence on the current spectrum because of the low intensity of the [Fe,C,3H]^+^ product.

When fragmentation occurred, up to 50% of the original [Fe,C,2H]^+^ intensity was retained, and the yield of Fe^+^ was roughly twice that of FeC^+^, as shown in Fig. S2a of the ESI.† This observation appears at odds with the calculated dissociation energies for the fragmentation channels of [M,C,2H]^+^ listed in Table 1, which indicate that formation of FeC^+^ and H_2_ is thermodynamically preferred. However, formation of Fe^+^ + CH_2_ requires direct cleavage of the Fe–C bond over a loose transition state (TS), whereas formation of FeC^+^ + H_2_ is more complicated as it requires cleavage of both C–H bonds and formation of the H_2_ bond, and thus likely involves a tight TS. Such a tight TS would make the latter process entropically less favorable, and further, it potentially could result in a barrier in excess of the endothermicity shown in Table 1.

The calculated ground state (GS) for [Fe,C,2H]^+^ is a *C*_2v_ symmetric iron carbene geometry, FeCH_2_^+^, with a ^4^B_1_ electronic state and a covalent double bond between the metal and CH_2_ group. Note that formation of this quartet spin species would require a spin surface crossing if the reactant Fe^+^ ions were efficiently cooled to their ^6^D ground state; however, it is also possible that the laser ablation source creates a population of the low-lying ^4^F excited state of Fe^+^. With the convention that the *z*-axis is the symmetry axis and that the molecule lies in the *yz* plane, the ^4^B_1_ GS has a Fe–C σ bonding molecular orbital (MO) (a_1_ symmetry, using 3d_*z*^2^_ on Fe, double occupancy), a Fe–C π bond (b_1_, Fe 3d_*xz*_, double), three non-bonding MOs localized on Fe: (a_1_, 3d_*x*^2^−*y*^2^_, double; a_2_, 3d_*xy*_, single; a_1_, 4s, single), and an antibonding MO combining the Fe 3d_*yz*_ and CH_2_ orbitals (b_2_, single). Thus, the valence MO occupation (excluding the four CH_2_ bonding electrons) is (1a_1_)^2^(1b_1_)^2^(2a_1_)^2^(1a_2_)^1^(3a_1_)^1^(1b_2_)^1^.

Several additional electronic states were found on the quartet spin surface that also have the symmetric *C*_2v_ structure. A second electronic state, ^4^B_2_, calculated to lie only 0.013 eV higher in energy, reverses the occupation of the 3d_*x*^2^−*y*^2^_ (2a_1_) and 3d_*xy*_ (1a_2_) MOs; their non-bonding character explains the small energy difference between these two states. In another low-lying state (^4^A_2_), one electron in the 3d_*x*^2^−*y*^2^_ (2a_1_) MO is transferred to the antibonding 1b_2_ MO, leading to decreased stability, 0.18 eV above the GS. Additional carbene structures were located for different spin states. A ^6^B_1_ state located 0.83 above the GS has a MO occupation of (1a_1_)^2^(1b_1_)^1^(2a_1_)^2^(1a_2_)^1^(3a_1_)^1^(1b_2_)^1^(2b_1_)^1^ where the 2b_1_ MO is the antibonding version of the 1b_1_ Fe–C π bond. Two carbene structures were found on the doublet surface, of which the lowest, ^2^A″ has the same electron configuration as the ^4^B_1_ ground state, (1a_1_)^2^(1b_1_)^2^(2a_1_)^2^(1a_2_)^1^(3a_1_)^1^(1b_2_)^1^, but is slightly distorted from *C*_2v_ symmetry (although the MOs can still be labeled using this symmetry) with Fe–H distances of 2.52 and 2.58 Å. This state lies 1.04 eV above the GS. The second doublet state located, a ^2^A_2_ (1a_1_)^2^(1b_1_)^2^(2a_1_)^2^(1a_2_)^1^(3a_1_)^0^(1b_2_)^2^ state with *C*_2v_ symmetry, lies only 0.02 eV above the ^2^A″ state. A summary of all structures and electronic states, including energies relative to the reactants, found in this study is presented in the ESI,† Table S1.

We also explicitly checked whether stable agostic iron carbene geometries could be located, but as there are no empty 3d orbitals on Fe^+^, these all converged to symmetric carbene geometries. We did find a hydrido iron carbyne structure, HFeCH^+^ but only on the sextet surface (^6^A″) lying 3.32 eV above the GS. The lowest energy species with both hydrogen atoms connected to iron is a dihydrogen iron carbide, (H_2_)FeC^+^ (^2^A′) at 2.70 eV. Here, the H–H bond distance of 0.787 Å is slightly longer than the 0.743 Å calculated here at the same level of theory for the free H_2_ molecule. A dihydrido iron carbide, H_2_FeC^+^ (H–H distance is 2.88 Å) in a ^4^A″ state was found 5.54 eV above the GS.

Fig. 1b–h shows the simulated IR spectra of different [Fe,C,2H]^+^ isomers and states located computationally. Many species shown have calculated vibrational modes near the experimental bands. Of the six fundamental vibrations of *C*_2v_ metal carbenes, two correspond to the symmetric and antisymmetric C–H stretch vibrations and are calculated to lie near 3100 cm^−1^. Four modes are located somewhere between 500 and 1500 cm^−1^: the CH_2_ rocking mode (in-plane CH_2_ bend, b_2_ symmetry, b-type transition), the M–C stretch (a_1_, a-type), the CH_2_ wagging mode (out-of-plane CH_2_ bend, b_1_, c-type), and the CH_2_ scissoring mode (a_1_, a-type). For the FeCH_2_^+^ (^4^B_1_) GS structure, these modes are calculated at 554, 627, 787, and 1321 cm^−1^, respectively, Table 2, with the C–H stretches at 3078 and 3182 cm^−1^. According to B3LYP calculations, the CH_2_ wagging and rocking vibrations are most intense (36 and 101 km mol^−1^), whereas the Fe–C stretch and CH_2_ scissoring mode (both involving a-type transitions) have calculated intensities of only 5 and 1 km mol^−1^, respectively. The effects of varying the MO occupation on the calculated IR spectrum are only marginal, with the main difference being a shift of the CH_2_ out-of-plane wagging vibration from 787 cm^−1^ (^4^B_1_ GS) to 716 cm^−1^ (^4^B_2_), Table 2.

**Table 2:** Experimental band positions and strengths (s = strong, m = medium, w = weak) accompanied by theoretical calculated frequencies, intensities, and descriptions for the assigned structures

	Experiment		Theory B3LYP/CCSD^a^		Mode description
	Frequency (cm^−1^)	Strength	Frequency (cm^−1^)	Intensity (km mol^−1^)	
FeCH_2_^+^ (^4^B_1_)			554/570	36/45	CH_2_ rocking
	632	s	627/647	5/43	M–C stretch
	767	m	787/754	101/106	CH_2_ wagging
	—	—	1321/1358	1/1	CH_2_ scissor
FeCH_2_^+^ (^4^B_2_)			555/567	40/50	CH_2_ rocking
	632	s	632/646	9/43	M–C stretch
	767	m	716/744	69/98	CH_2_ wagging
	—	—	1319/1359	0/1	CH_2_ scissor
CoCH_2_^+^ (^3^A_2_)			622/625	36/41	CH_2_ rocking
	673	s	631/690	0/25	M–C stretch
	811	s	877/861	88/100	CH_2_ wagging
	948	m	1377/1410	3/23	CH_2_ scissor
CoCH_2_^+^ (^3^A_1_)			601/624	31/39	CH_2_ rocking
	673	s	625/693	1/14	M–C stretch
	811	s	893/881	89/100	CH_2_ wagging
	948	m	1382/1416	3/30	CH_2_ scissor

a B3LYP and CCSD calculations used the def2-TZVPPD basis set.

The rotational profile simulations for all species in Fig. 1 reflect the large rotational constants involved (*e.g.*, 9.98, 0.39, and 0.38 cm^−1^ for the ^4^B_1_ GS), which, in combination with the room temperature conditions of the experiment, lead to broad bands with complex substructure. The exothermicity of the reaction of Fe^+^ with oxirane is 0.16 ± 0.04 eV, indicating that the three lowest energy states are accessible at thermal energies (and potentially others if appreciable electronic excitation were retained by Fe^+^). Indeed, the energy difference between the ^4^B_1_ and ^4^B_2_ states is so small that population of both states is reasonable. Nevertheless, none of the three low-energy species in panels b–d yields a very convincing match with the experiment, although for all three structures, a broad absorption is predicted with the center of gravity close to the center of the experimentally observed band.

Comparison between simulated spectra for higher energy species and the experimental spectrum does not offer reason to suspect their presence. The ^6^B_1_ structure at +0.83 eV has a significantly different IR spectrum, most notably from the intensity increase of the CH_2_ scissoring mode to 58 km mol^−1^, while the CH_2_ wagging mode massively red shifts to 142 cm^−1^ and is left with an intensity of 14 km mol^−1^. The only spectrum that shows a compact band structure is that calculated for the ^2^A_2_ species, which arguably resembles the experimental spectrum best in shape but is shifted to the blue; it is, however, difficult to see how the formation of a structure at more than 1 eV above the GS could be favored, especially given the number of spin crossings required for its formation.

### Alternative levels of theory for the spectroscopy of FeCH_2_^+^

**Fig. 2:**
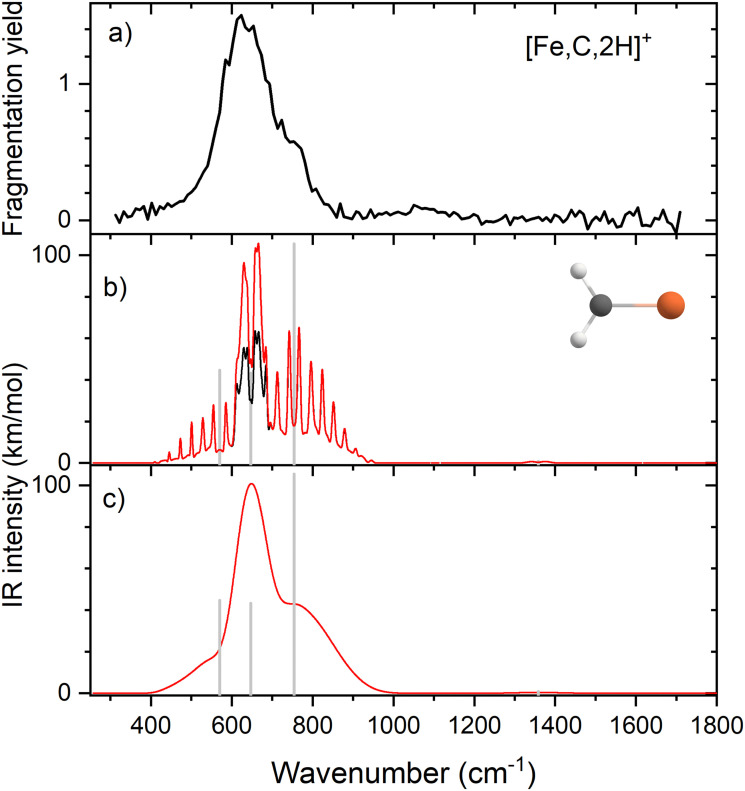
(a) Experimental IRMPD spectrum of [Fe,C,2H]^+^; (b) harmonic stick spectrum of the FeCH_2_^+^ (^4^B_1_) structure at the CCSD/def2-TZVPPD level and rovibrational simulations using the original IR intensity for the Fe–C stretch mode at 43 km mol^−1^ (black) and with a double value (red); (c) same as (b) but convolved with a 9% FWHM Gaussian lineshape function to simulate broadening effects.

Because of the mismatch in the comparison of the B3LYP computational spectra with experiment, we also explored other levels of theory, starting with MP2. The MP2 calculation of the ^4^B_1_ state provided similar vibrational frequencies as the B3LYP calculation, 593, 660, 710, 1409, 3160, and 3273 cm^−1^, but to our surprise, the intensity profile was substantially different, primarily because the intensities of the two a-type transitions were much higher, 76 and 34 km mol^−1^, respectively, *versus* 5 and 1 km mol^−1^ for B3LYP (see Fig. S4, ESI†). The enhanced intensity of the Fe–C stretch would be sufficient to better reproduce the experimental band, whereas the enhanced intensity of the CH_2_ scissors mode is inconsistent with the failure to observe a band in this wavenumber range. To explore this phenomenon further, we performed CCSD/def2-TZVPPD calculations. CCSD(T)//CCSD calculations place the ^4^B_2_ state only 0.0005 eV above the ^4^B_1_ GS. CCSD geometry optimizations for the ^4^B_1_/^4^B_2_ states provide similar frequency predictions as the B3LYP results, 570/567, 647/646, 754/744, 1358/1359, 3122/3122, and 3228/3227 cm^−1^, but again the intensity profile is distinct. Now, the CH_2_ rock (b-type) and wag (c-type) have similar intensities (45/50 and 106/98, respectively), whereas the a-type Fe–C stretch has a much larger predicted intensity (43/43 km mol^−1^) compared to the B3LYP results. Unlike the MP2 results, the scissors (a-type) mode has an intensity (<1/<1 km mol^−1^) consistent with experiment. Fig. 2 compares the CCSD predicted spectrum for the ^4^B_1_ GS (the ^4^B_2_ spectrum is very similar, see Fig. S5b of the ESI†) with the experimental spectrum and clearly provides an improved match compared with the B3LYP results of Fig. 1. Fig. 2 also includes a predicted spectrum with the Fe–C stretch band having an intensity that matches the MP2 calculation. As shown in Fig. 2c that includes more broadening, the latter is probably the best match to the experimentally observed band. It can also be realized that some of variations in intensities may be a result of comparing the multiple photon experimental absorptions with the single photon theoretical predictions.

**Fig. 3:**
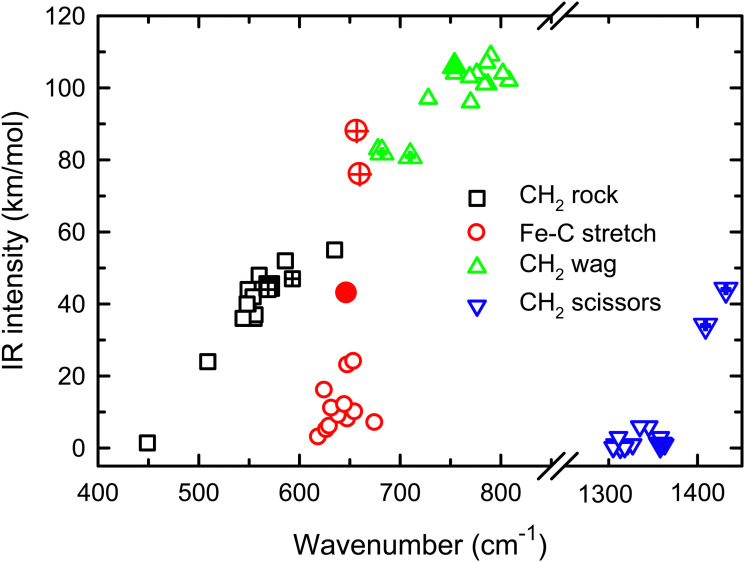
Theoretical IR intensities *versus* wavenumber for the four vibrations accessible experimentally in the present experiments. CCSD results are closed symbols and MP2 results are hatched.

The enhanced intensity in the Fe–C stretch in both MP2 and CCSD computations is the result of a well-known phenomenon in DFT calculations,^43^ which generally underestimate the charge separation in something like the Fe–CH_2_ bond and therefore the dipole derivative with respect to the Fe–C stretch. For instance, in the ^4^B_1_ ground state, the charges on Fe and CH_2_ calculated (using the natural bond orbital formalism)^44^ at the equilibrium geometry using B3LYP are +1.16 *e* and −0.16 *e*, respectively, (for a charge difference of 1.32 *e*), but +1.51 *e* and −0.51 *e* for CCSD (charge difference of 2.01 *e*). As the bond extends by 0.2 Å, the charge difference changes by +0.02 *e* for B3LYP (leading to very low IR intensity) but by −0.15 *e* for CCSD (yielding a moderate IR intensity). This phenomenon was explored further by examining results for several other density functionals,^35,36,45–55^ as detailed in Table 3. In general, all DFT approaches show the same trends with the predicted intensity of the Fe–C stretch varying from 3–24 km mol^−1^, while maintaining the CH_2_ wag at 96–109 km mol^−1^ (except M06-2X at 83 km mol^−1^). All of these lie less than about half the intensity found at the CCSD level. The predicted frequency for the Fe–C stretch is insensitive to the level of theory, varying from 619 to 675 cm^−1^, which indicates that the different levels of theory yield similar force constants for this motion. In contrast, the CH_2_ wag shows modest dispersion, 678–808 cm^−1^. This is shown pictorially in Fig. 3 for all four modes accessible in the present experiment. The Fe–C stretch shows the largest range of intensities with the DFT values clustered at low values, CCSD being intermediate, and MP2 relatively high. The CH_2_ wags have comparable intensities for all levels of theory. The dispersion in intensities is also generally small for the CH_2_ rock and scissors motions. Here, the outliers for the CH_2_ rock are MN15L (1 km mol^−1^) and TPSS (24 km mol^−1^), with all other levels of theory clustered between 36 and 55 km mol^−1^. Note that these two levels also predict frequencies much lower than other levels. For the CH_2_ scissors, the outliers are MP2 harmonic (34 km mol^−1^) and anharmonic (44 km mol^−1^) with all other levels of theory, including CCSD, predicting a very weak band intensity. Here, most levels of theory predict a frequency between 1313 and 1358 cm^−1^, whereas the MP2 calculations shift this up by more than 50 cm^−1^. Because of the disparity in the predicted intensities of the M–C stretch, we treat its intensity as a potential adjustable parameter in further comparisons between theory and experiment. The experimentally observed band positions, together with harmonic normal mode frequencies and intensities calculated at the B3LYP and CCSD levels are combined in Table 2.

**Table 3:** Predicted vibrational frequencies in cm^−1^ (intensities in km mol^−1^) for FeCH_2_^+^ (^4^B_1_) calculated at multiple levels of theory

Level of theory	CH_2_ rock	Fe–C stretch	CH_2_ wag	CH_2_ scissors
TPSS^46^	509 (24)	619 (3)	808 (102)	1313 (0)
B3LYP^35,36^	554 (36)	627 (5)	787 (101)	1321 (1)
B3LYP-GD3BJ^47^	555 (36)	627 (5)	787 (101)	1321 (1)
X3LYP^36,48^	556 (37)	630 (6)	784 (101)	1321 (1)
ωB97X-D^49^	549 (44)	675 (7)	790 (109)	1345 (6)
PW6B95D3^50^	549 (40)	638 (8)	776 (104)	1327 (1)
B3PW91^35,45^	544 (36)	639 (9)	769 (103)	1307 (1)
Cam-B3LYP^51^	554 (42)	655 (10)	786 (107)	1335 (6)
MN15L^52^	449 (1.4)	632 (11)	770 (96)	1318 (<1)
PBE0^53^	548 (40)	645 (12)	754 (104)	1305 (<1)
M06-2X^54^	560 (48)	625 (16)	678 (83)	1311 (3)
BHHLYP^36^	586 (52)	648 (23)	728 (97)	1358 (3)
PBE0-DH^55^	635 (55)	654 (24)	802 (104)	1363 (1)
CCSD(full)^41^	570 (45)	647 (43)	754 (106)	1358 (1)
MP2(full)^40^	593 (47)	660 (76)	710 (81)	1409 (34)
MP2(full) – anharmonic	569 (44)	657 (88)	682 (82)	1432 (44)

### IR spectroscopy of CoCH_2_^+^

Resonant IR irradiation of [Co,C,2H]^+^ (*m*/*z* = 73) leads to two photofragments, Co^+^ and CoC^+^, with Co^+^ being the major fragment (∼90%), as shown in Fig. S2b of the ESI.† This result is consistent with the thermochemistry in Table 1. Similar to the Fe case, the calculated dissociation energies for the fragmentation channels of [Co,C,2H]^+^ listed in Table 1 slightly favor formation of CoC^+^ and H_2_. As discussed above, the observation that Co–CH_2_ cleavage dominates may be because dehydrogenation is entropically less favorable and could have a barrier in excess of the endothermicity shown in Table 1.

**Fig. 4:**
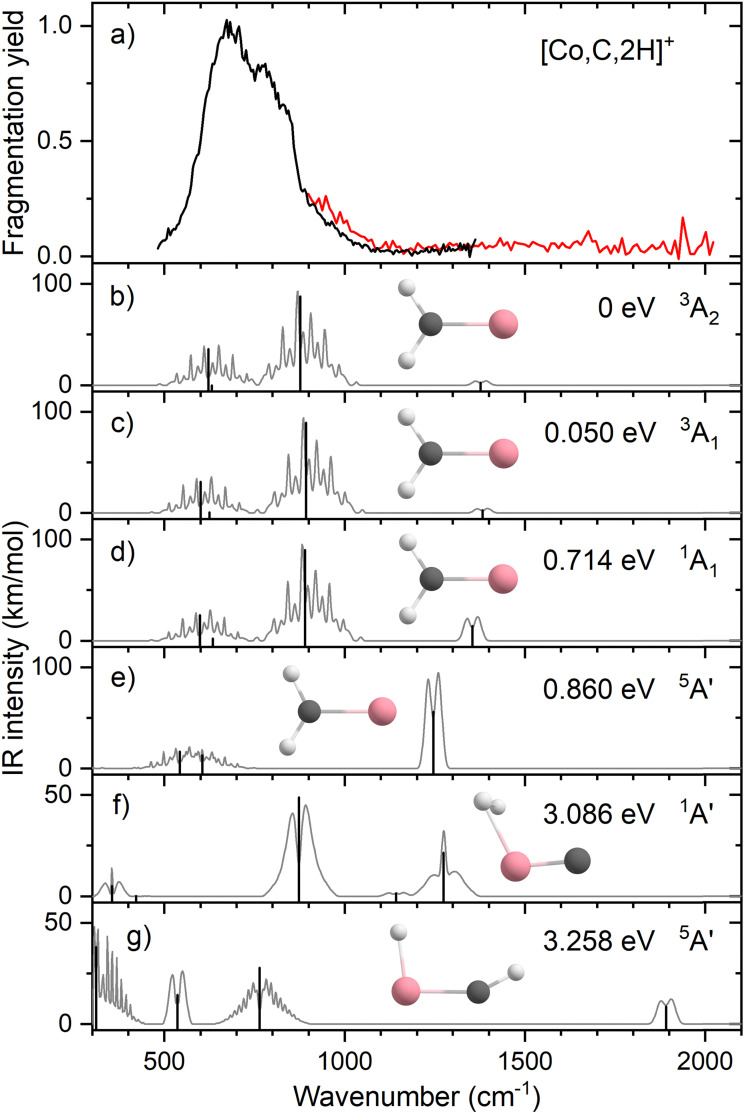
(a) Experimental IRMPD spectrum of [Co,C,2H]^+^, the black trace is the average of two scans, the red trace is a single scan; (b)–(g) B3LYP/def2-TZVPPD calculated IR spectra of different [Co,C,2H]^+^ states and isomers with the harmonic vibrations in black and the rovibrational envelopes in grey accompanied by geometric structure, relative energy, and electronic state.

Fig. 4a shows the experimental IRMPD spectrum of [Co,C,2H]^+^. It shows one dominant band, which appears to consist of two maxima around 673 and 811 cm^−1^. The band has a relatively long tail to the blue which could be a third, weaker band. Its center is estimated around 948 cm^−1^. Fig. 4b–g shows the B3LYP simulated IR spectra of different [Co,C,2H]^+^ isomers and states. The ground state is a symmetric cobalt carbene geometry, CoCH_2_^+^, with a ^3^A_2_ electronic state. The MOs are the same as for FeCH_2_^+^, such that the valence MO configuration for the ^3^A_2_ state is (1a_1_)^2^(1b_1_)^2^(2a_1_)^2^(1a_2_)^1^(3a_1_)^1^(1b_2_)^2^, with the singly occupied MOs being the 3d_*xy*_ (1a_2_) and 4s (3a_1_) MOs. A ^3^A_1_ state, only 0.050 eV higher in energy, is obtained by moving an electron from the 3d_*x*^2^−*y*^2^_ (2a_1_) MO to the 3d_*xy*_ (1a_2_) MO, both non-bonding. Thus, the ^3^A_2_ and ^3^A_1_ states of CoCH_2_^+^ are analogous to the ^4^B_1_ and ^4^B_2_ states of FeCH_2_^+^ with addition of the extra electron to the antibonding 1b_2_ MO. If the 1b_2_ MO is singly occupied instead of the 3a_1_ or 1a_2_ MOs, ^3^B_1_ and ^3^B_2_ states are found and lie 0.41 and 0.90 eV above the GS. Singlet spin states of CoCH_2_^+^ (^1^A_1_ and ^1^A_2_) were found 0.71 and 0.77 eV above the GS. These states have the same valence MO configuration as the triplet states, but the two singly occupied MOs are singlet coupled instead of triplet coupled, leading to the higher energy. Similar to [Fe,C,2H]^+^, trial structures for agostic cobalt carbenes collapsed into the symmetric carbenes, again because there are no empty 3d orbitals to support this distorted structure. The lowest energy quintet spin species is a distorted carbene with a ^5^A′ state found at 0.86 eV with one of the hydrogen atoms closer to the cobalt atom (2.56 and 2.63 Å). A second quintet carbene (^5^A″) was found 0.94 eV above the GS and has both hydrogen atoms bent slightly out of the molecular plane (H–H–Co–C dihedral angle of 7.3°). The lowest-energy hydrido cobalt carbyne, HCoCH^+^, was found on the quintet surface (^5^A′) at 3.26 eV above the GS. All geometries with two hydrogens bound to cobalt converged to dihydrogen cobalt carbides, (H_2_)CoC^+^, with the lowest being a ^1^A′ state with an energy 3.09 eV above the GS. Table S1 of the ESI† lists all [Co,C,2H]^+^ species located theoretically.

In contrast to the spectra calculated for Fe carbene isomers, those for cobalt carbene states are very much alike. As for the analogous FeCH_2_^+^ states, the ^3^A_2_ and ^3^A_1_ states of CoCH_2_^+^ shift the CH_2_ rocking frequency, from 622 to 601 cm^−1^, respectively, and the CH_2_ wagging mode, from 877 to 893 cm^−1^. The intensities of the CH_2_ rocking and CH_2_ wagging modes differ by at most 16%. The Co–C stretch and CH_2_ scissoring modes both shift less than 5 cm^−1^ and have intensities below 3 km mol^−1^ in B3LYP calculations. Like the FeCH_2_^+^ results, the B3LYP spectra do not provide a good reproduction of the experimental spectrum for any structure located theoretically, Fig. 4.

**Fig. 5:**
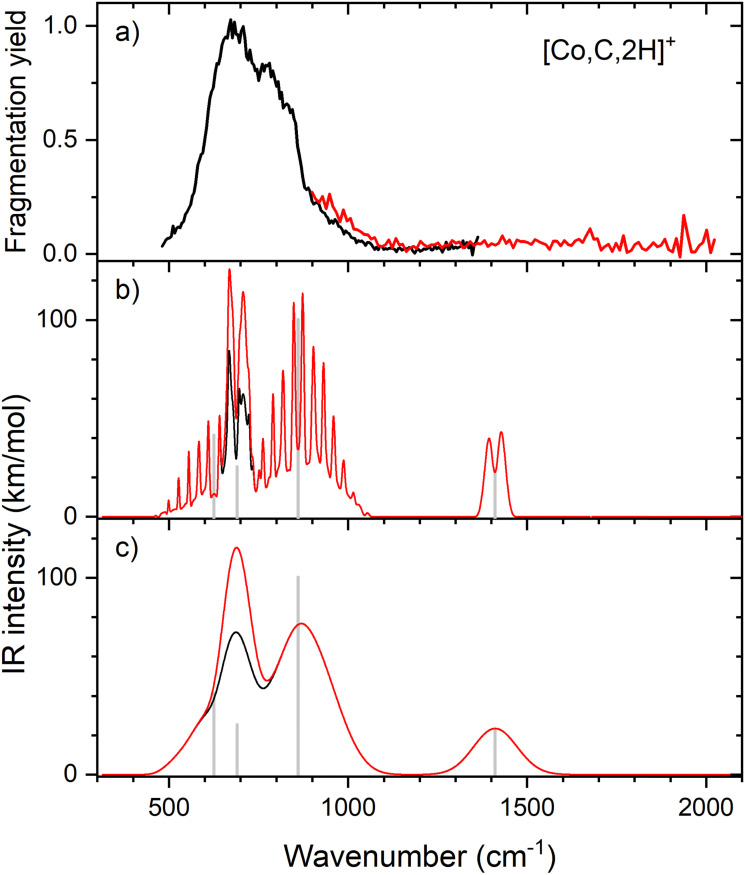
(a) Experimental IRMPD spectrum of [Co,C,2H]^+^; (b) harmonic stick spectrum of the CoCH_2_^+^ (^3^A_2_) structure at the CCSD/def2-TZVPPD level and rovibrational simulations using the original IR intensity for the Co–C stretch mode at 25 km mol^−1^ (black) and with a double value (red); (c) same as (b) but convolved with a 9% FWHM Gaussian lineshape function to simulate broadening effects.

Therefore, we again tested MP2 and CCSD approaches. Similar to FeCH_2_^+^, the intensity profile obtained from MP2 calculations (Fig. S4, ESI†) greatly enhances the Co–C stretch, such that it is slightly more intense than the CH_2_ out-of-plane wag (intensities of 76 and 83 km mol^−1^ for the ^3^A_2_ and ^3^A_1_ states, respectively). CCSD(T)//CCSD calculations indicate that the ^3^A_1_ state lies only 0.015 eV above the ^3^A_2_ GS. Fig. 5 shows the spectrum predicted by the CCSD level of theory for the ^3^A_2_ GS, where the Co–C stretch has a predicted intensity of 25 km mol^−1^ (the spectrum for the low-lying ^3^A_1_ state is very similar as shown in Fig. S5d of the ESI†). As for the case of FeCH_2_^+^, the experimental spectrum is matched better if the intensity of this band is approximately twice that predicted at the CCSD level. Here, the moderate IR intensity (23 km mol^−1^) predicted for the CH_2_ scissor vibration is not matched by the observed spectrum. In any case, we attribute the experimental spectrum to a combination of the symmetric cobalt carbene cation in both the closely-spaced ^3^A_2_ and ^3^A_1_ states because the reaction of ground state Co^+^ (^3^F, 3d^8^) with oxirane is slightly endothermic, by 0.08 ± 0.05 eV. Thus, formation of these states can be driven by near-thermal energies, as shown previously.^50^ Although contributions from electronically excited states could be present, the lowest energy excited state of Co^+^, ^5^D (4s^1^3d^7^), should be less reactive than the ^3^F (3d^8^) GS because the 4s orbital is occupied.^56^

Clearly, although the CCSD level reproduces the experimental spectrum with reasonable fidelity in the 500–1200 cm^−1^ range, the predicted peak near 1400 cm^−1^ (CH_2_ scissors) is not found in the experimental spectrum. When irradiating [Co,C,2H]^+^ under higher IR fluences (not shown), we observed a broad, unresolved band stretching from 500–1800 cm^−1^. We speculate that under the current experimental conditions, IR excitation is not efficient enough to induce fragmentation at the predicted 1400 cm^−1^ band and that higher intensities rapidly lead to non-resonant absorption.

### Group 8 and 9 trends: carbene verses hydrido carbyne

**Fig. 6:**
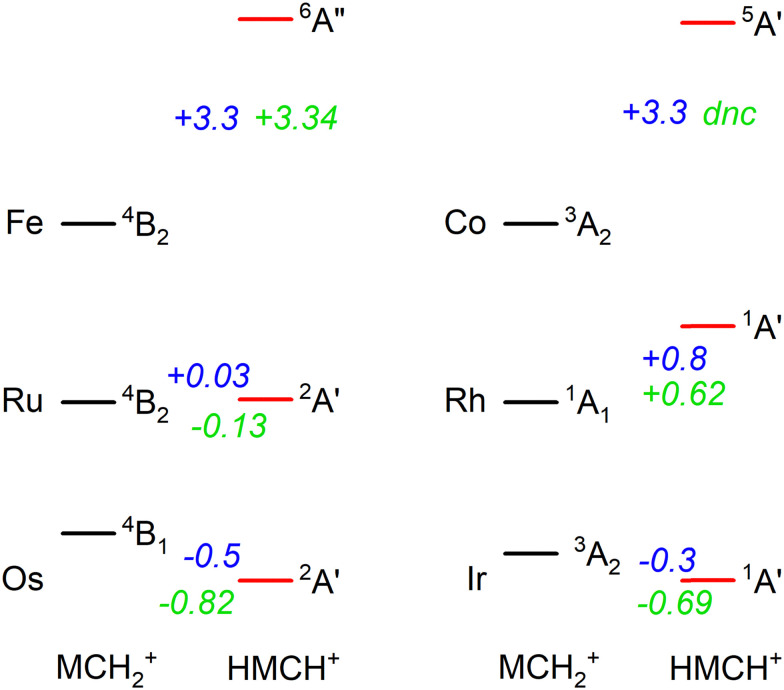
Energetic comparison between the lowest energy species of carbene (MCH_2_^+^) and hydrido carbyne (HMCH^+^) structures for group 8 and 9 metal cations. For each, the lowest energy state of both configurations is indicated together with the energy difference, *E*(HMCH^+^) − *E*(MCH_2_^+^), in eV. B3LYP energetics for Os are taken from ref. 6, 11 and for Ir from ref. 8 the other values are from this work. B3LYP/def2-TZVPPD energies are shown in blue and CCSD(T)//CCSD energies are in green. CCSD calculations for HCoCH^+^ on the quintet spin surface did not converge (dnc).

In previous work, we have presented the experimental IRMPD spectra of [Ru,C,2H]^+^, [Rh,C,2H]^+^,^20^ [Os,C,2H]^+^, and [Ir,C,2H]^+^,^8–11^ the 4d and 5d congeners of the group 8 and 9 species presented in this study. If we put the current assignments for Fe and Co in perspective with the previous findings, we note that both 5d elements (Os and Ir) prefer the formation of a hydrido carbyne structure, whereas the 3d elements form carbene structures. Our recent work showed that the 4d elements are mixed, with Ru forming both structures and Rh yielding only the carbene.^20^ Energetically, 5d hydrido carbynes are preferred, but more strongly so for the group 8 elements as indicated by the energetics presented in Fig. 6. This is consistent with the assignment of the product of osmium (a group 8 element) to HOsCH^+^ (^2^A′), whereas part of the product for iridium (group 9) is assigned to a carbene structure (0.3 eV higher in energy). This trend appears to hold for 4d elements, where the ^4^B_2_ ruthenium carbene is almost isoenergetic with the ^2^A′ hydrido ruthenium carbyne, whereas the HRhCH^+^ (^1^A′) is significantly higher in energy than the RhCH_2_^+^ (^1^A_1_). For 3d elements, hydrido carbyne structures are not competitive at all, lying several eV higher in energy. Thus, as one moves down a group in the periodic table, hydrido carbyne structures become increasingly competitive in terms of energy compared to carbene structures, but less so along a row.

One rationale for these trends is thermodynamic. H–M≡CH^+^ has four covalent bonds to the metal whereas MCH_2_^+^ only has two covalent M=C bonds. In general, the 5d TM have stronger bonds to C and H than the 4d or 3d TM, *e.g.*, for group 9, see Fig. 4 in ref. 4 (for group 8, see ref. 6, 19, 22, 24, 57 and 39). Thus, the more highly coordinated hydrido carbyne structure becomes more favorable for 5d relative to 4d TMs and even more so for 3d TMs. In addition, one can also consider the promotion energies required to put the metal cation into an electronic configuration needed for bonding. (Here, the promotion energies are averaged over all spin–orbit levels and taken from ref. 58.) For the group 8 elements, both MCH_2_^+^ and HMCH^+^ species correlate with an (*n* − 1)d^6^*n*s^1^ electronic configuration on M^+^ so the thermodynamic considerations dictate the vertical trends. For the group 9 elements, the triplet states of MCH_2_^+^ correlate with the (*n* − 1)d^7^*n*s^1^ electronic configuration on M^+^. This is the ground state (GS) for Ir^+^, a low-lying state for Co^+^ (0.43 eV above the ^5^F, 3d^8^ GS), but a high-energy state for Rh^+^, 2.13 eV higher in energy than the ^5^F (4d^8^) GS. Thus, the RhCH_2_^+^ prefers the ^1^A_1_ state that correlates with the ^5^F (4d^8^) GS. For the HMCH^+^ structures of the group 9 elements, the ^1^A′ GS correlates with either an (*n* − 1)d^6^*n*s^2^ or low-spin (*n* − 1)d^7^*n*s^1^ (^3^F) electronic configuration on M^+^. The former configuration is generally higher in energy, whereas the latter lies 1.21, 3.13, and 1.33 eV above the GS of Co^+^, Rh^+^, and Ir^+^, respectively. Because these promotion energies are much higher than those for the group 8 metal cations, the HMCH^+^ structures are more favorable for group 8 than for group 9, as seen in Fig. 6.

**Fig. 7:**
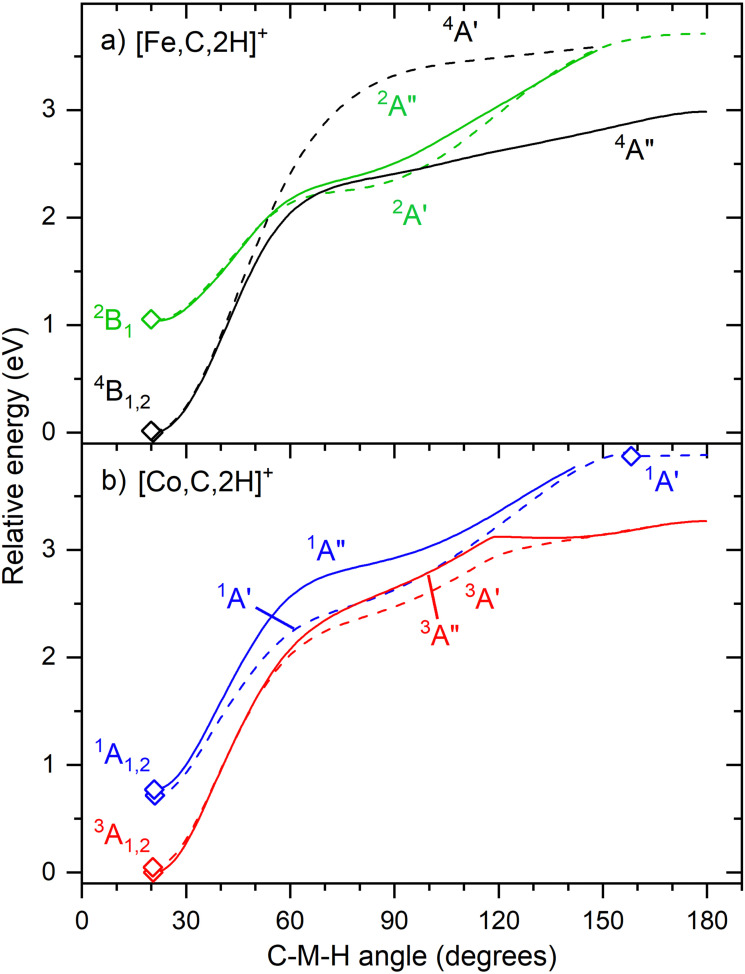
Potential energy surfaces of [M,C,2H]^+^ along the C–M–H angle calculated at the uB3LYP/def2-TZVPPD level of theory. Solid diamonds indicate calculated structures that successfully converged to true minima at the indicated C–M–H angle. Solid lines indicate A″ states while A′ states are indicated by dashed lines. The doublet spin surface is depicted in green, quartet in black, singlet in blue and triplet in red. All energy values are relative to the GS symmetric carbene structures.

Fig. 6 shows that the hydrido carbyne structures for 4d and 5d elements are found on the same or a lower spin surface as that of the lowest energy carbene structure. However, for Fe and Co, the lowest energy hydrido carbynes were found on the sextet and quintet spin surfaces, respectively. To investigate whether we might have missed carbyne structures on the lower spin surfaces, the potential energy surfaces (PESs) connecting the MCH_2_^+^ and HMCH^+^ structures were calculated for both metal cations investigated in this study (Fig. 7). PESs were generated by starting from a symmetric carbene structure and systematically increasing the C–M–H angle while the rest of the molecule was allowed to relax. The PES scans were generated for both the A′ and A″ configurations in dashed and solid lines, respectively. No stable minimum for a hydrido carbyne structure for Fe or Co is found on any of the two lowest spin surfaces, explaining why all trial hydrido carbyne geometries for [Fe,C,2H]^+^ and [Co,C,2H]^+^ converged into the carbene structure. For Co, a very shallow well was located along the ^1^A′ surface, but this species is 3.7 eV above the symmetric cobalt carbene ground state.

### Group 8 and 9 trends: charge separation

The observation that the intensities of the M–C stretches of FeCH_2_^+^ and CoCH_2_^+^ are not predicted well at DFT levels of theory leads one to wonder how extensive this particular problem is. As noted above, our work with the 5d TM systems utilized B3LYP calculations that reproduced the experimental spectra with fidelity. Likewise, our recent studies of [Ru,C,2H]^+^ showed comparable predictions between B3LYP and CCSD levels of theory.^20^ For [Rh,C,2H]^+^, the situation was complicated by the multireference character of the RhCH_2_^+^ states such that more advanced CCSD methods supplied the best reproduction of the data. Here, we directly compare the B3LYP and CCSD predictions for the group 8 and 9 transition metals to see how pervasive the problem is. We find that for the ^4^B_1_ states of the group 8 carbenes, the only mode that changes intensity drastically (factor of 8 or more) is the M–C stretch for FeCH_2_^+^. For both RuCH_2_^+^ and OsCH_2_^+^, the intensity of the M–C stretch remains very small. For the group 9 carbenes in the ^3^A_2_ state, CoCH_2_^+^ shows a dramatic increase in intensity for the M–C stretch and a more modest increase for the CH_2_ scissors, whereas for RhCH_2_^+^ and IrCH_2_^+^, the M–C stretch intensities are comparable for B3LYP and CCSD calculations and the CH_2_ scissors changes by factors of less than three. The results are summarized in Table S5 of the ESI.† Thus, the failure of DFT in predicting the intensities of the M–C stretch appears to be confined to the first-row (3d) TM.

## Conclusion

Reacting the 3d group 8 and 9 transition metal ions (M^+^ = Fe^+^ and Co^+^) with oxirane in a room temperature ion trap led to the formation of [M,C,2H]^+^. These species were mass isolated in the FTICR mass spectrometer coupled to the infrared intracavity free-electron laser FELICE, where they were spectroscopically characterized. The IRMPD spectra of [M,C,2H]^+^ with M = Fe and Co each contain one broad band around 700 cm^−1^ with minor differences in the extent of substructure. On the basis of B3LYP and CCSD(T)//CCSD energetics, both structures are expected to be carbenes. Comparison of the experimental IRMPD spectra with the predictions from DFT calculations offer poor agreement, making the assignment of structures tenuous at best. Calculations at the CCSD level offer a large improvement in the predicted intensities for the M–C stretch vibrations that match the experimental spectrum much better, although for CoCH_2_^+^, a simultaneous increase in the CH_2_ scissor mode intensity is not matched by experiment. Nevertheless, these calculations suggest the structures of [M,C,2H]^+^ for M = Fe and Co are indeed carbenes, each in two nearly degenerate electronic states.

The experimental spectra for [Fe,C,2H]^+^ and [Co,C,2H]^+^ are reproduced much better by CCSD calculations than by B3LYP calculations, primarily because the predicted intensity of the M–C stretch is much higher according to CCSD calculations. Indeed, enhanced M–C stretch intensities (consistent with MP2 calculations) provide even better agreement with experiment. This failure of the B3LYP functional appears to be systematic for all DFT approaches, although some functionals yield improved intensities but still do not match the CCSD predictions. This appears to be a consequence of a reduced separation in charge in the MCH_2_^+^ species. Exploration of this effect as one moves down the periodic table indicates that this failure does not propagate from the 3d TM to the 4d and 5d TM, where DFT approaches provide spectra that are in good agreement with experiment. Further, we explore the relative stabilities of the MCH_2_^+^*versus* HMCH^+^ species in terms of the periodic trends for these group 8 and 9 transition metals: Fe, Ru, Os and Co, Rh, Ir. It appears that promotion energy arguments are involved coupled with the number and strength of the covalent bonds needed to form the two structures.

## Conflicts of interest

There are no conflicts to declare.

## Supplementary Material

CP-026-D4CP00026A-s001
