# Supplementary material for: IR spectroscopic characterization of 3d transition metal carbene cations, FeCH2+ and CoCH2+: periodic trends and a challenge for DFT approaches
Source: Phys Chem Chem Phys. 2024 Feb 7;26(13):9948–62. doi: 10.1039/d4cp00026a (PMC10968520; doi:10.1039/d4cp00026a)
Supplement: CP-026-D4CP00026A-s001 [file CP-026-D4CP00026A-s001.pdf]

Electronic Supplementary Material (ESI) for Physical Chemistry Chemical Physics.
This journal is © the Owner Societies 2024

IR spectroscopic characterization of 3d transition metal carbene cations, $FeCH_2^+$ and $CoCH_2^+$: Periodic Trends and A Challenge for DFT approaches

Frank J. Wensink,[1] Corry E. Smink,[1] P. B. Armentrout,[2] and Joost M. Bakker[1]

[1]Radboud University, Institute for Molecules and Materials, FELIX Laboratory, Toernooiveld 7, 6525 ED Nijmegen, The Netherlands
[2]University of Utah, Department of Chemistry, 315 South 1400 East, Salt Lake City, Utah 84112, United States

Supporting Information

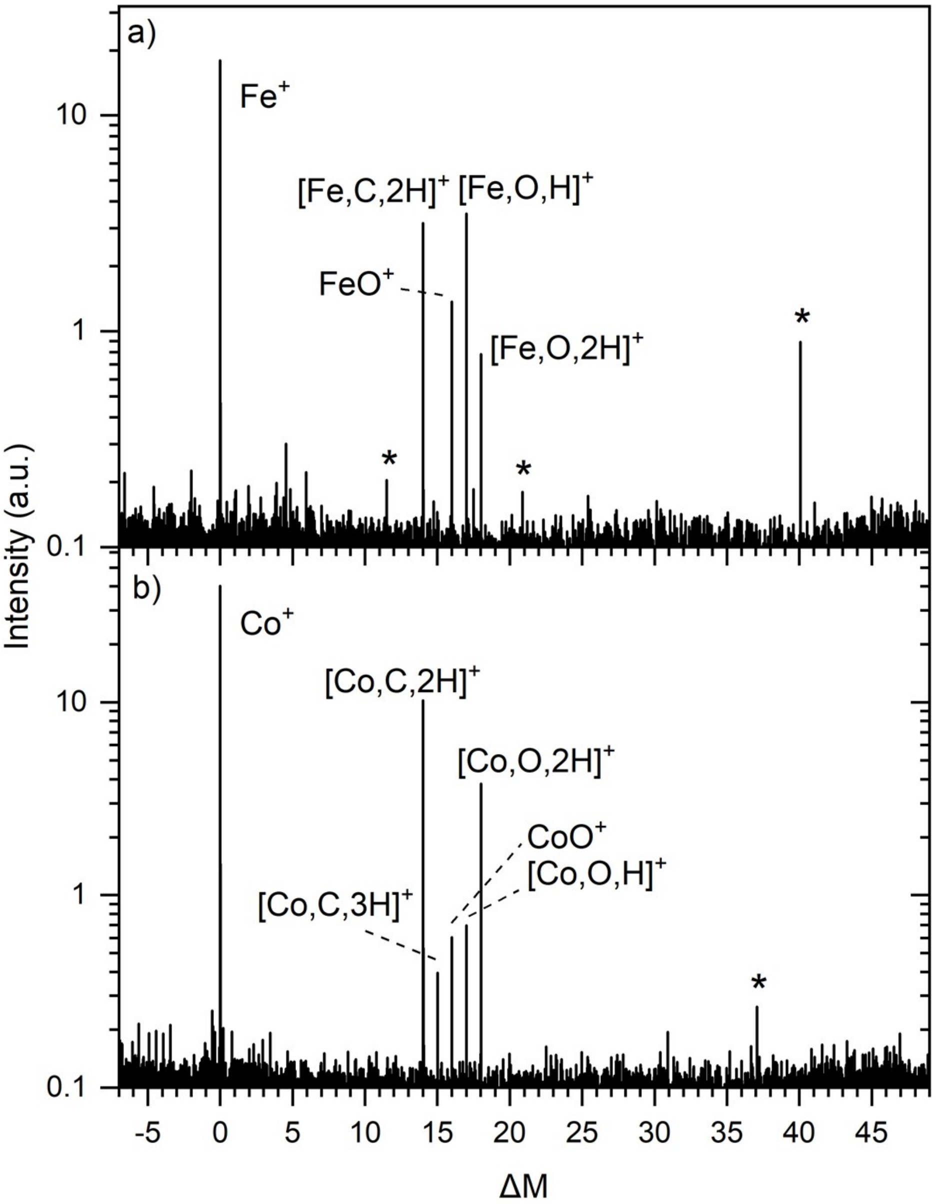

Figure S1: Product mass distributions of the reaction between ethylene oxide and a) Fe^+ ($\Delta M = m/z - 55.937$) and b) Co^+ ($\Delta M = m/z - 58.935$). Some artifacts resulting from electrical noise pickup are denoted by an asterisk (*).

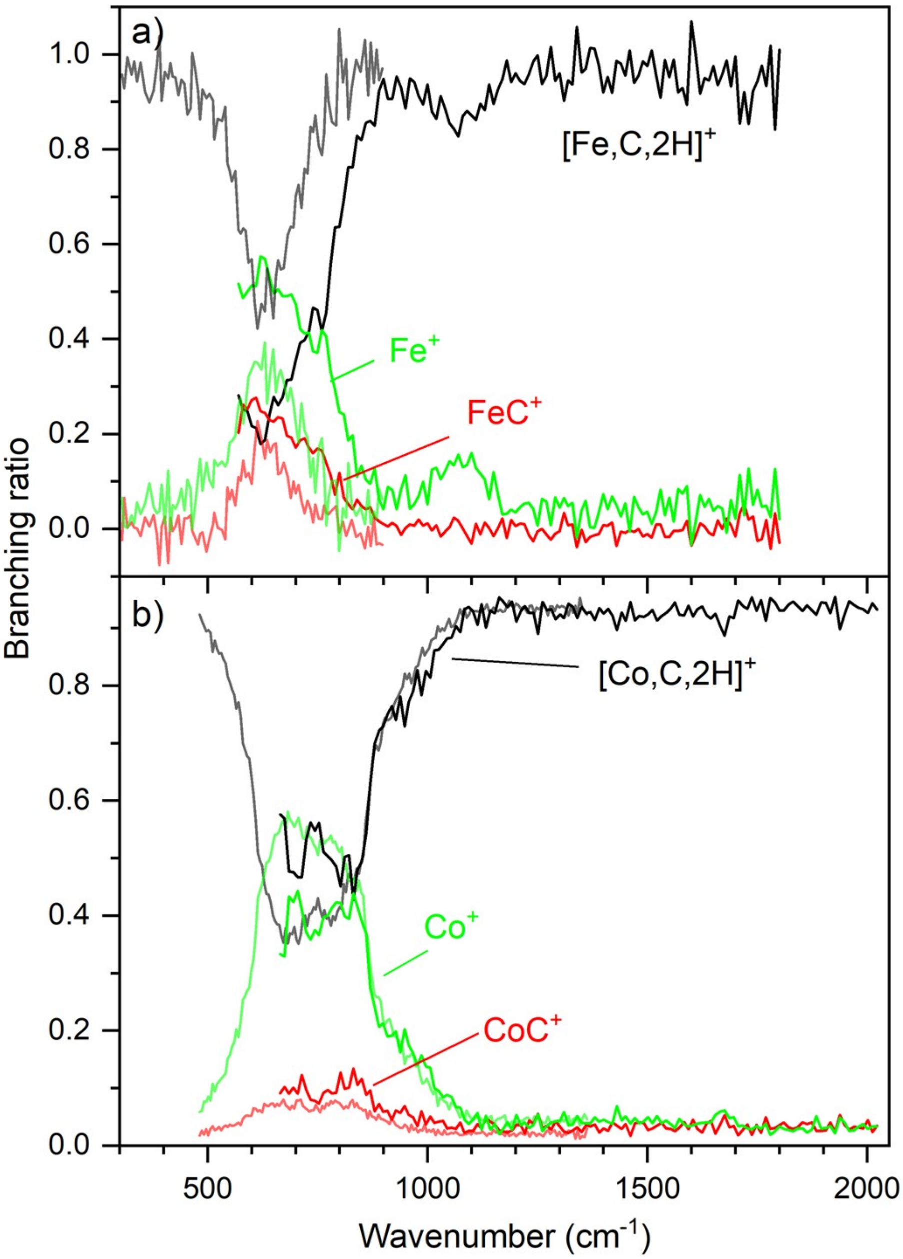

Figure S2: Observed ion intensities of $[M,C,2H]^+$ (black trace), MC^+ (red) and M^+ (green) normalized to their sum as a function of IR wavenumber for M = Fe (panel a) and Co (panel b). Bright versus light colors indicate measurements with different FEL settings. The signal increase in the Fe^+ channel around 1100 cm^{-1} is attributed to fragmentation of an unwanted $[Fe,C,3H]^+$ reaction product that was not mass-ejected prior to irradiation.

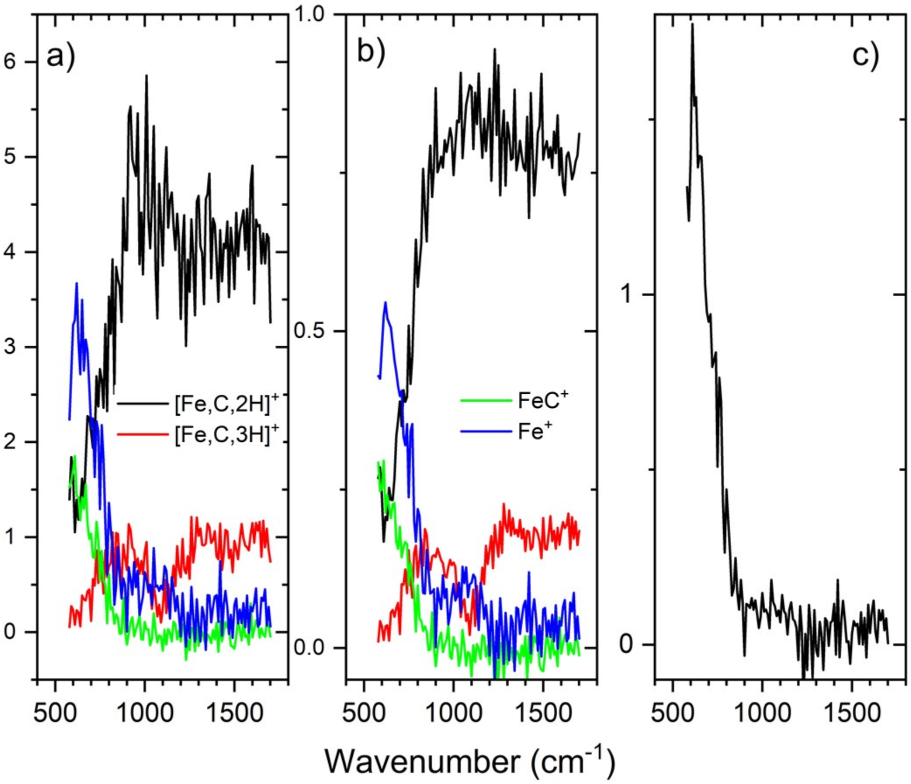

Figure S3: Observed ion intensities of $[M,C,2H]^+$ (black trace), [MC,3H] (red), MC^+ (green) and M^+ (blue) during one single scan (panel a), and each channel normalized to the sum of all four (panel b); resulting IR fragmentation yield for $[Fe,C,2H]^+$ calculated using Fe^+ and FeC^+ as fragments.

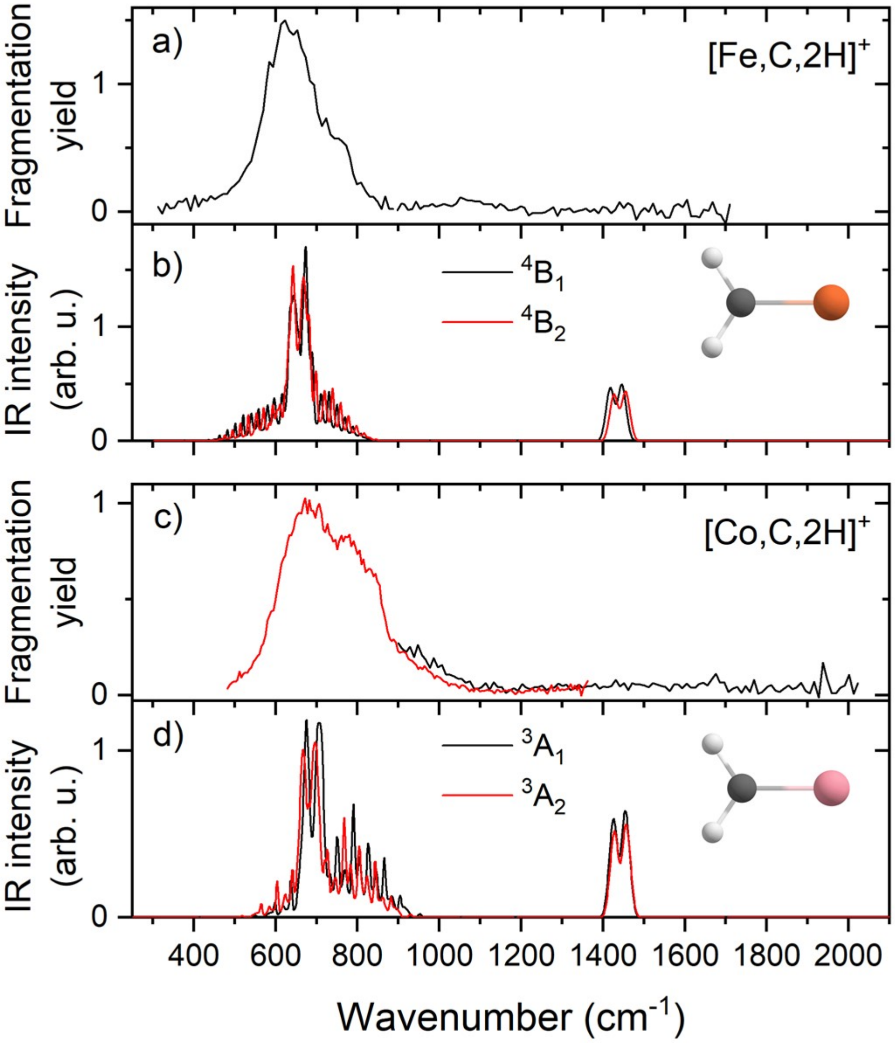

Figure S4: Experimental IRMPD spectra of $[Fe,C,2H]^+$ (panel a) and $[Co,C,2H]^+$ (panel c). Comparison of the spectra calculated at the MP2(full)/def2-TZVPPD level (panel b) $FeCH_2^+$ ($^4B_1$, black) and ($^4B_2$, red) and (panel d) $CoCH_2^+$ ($^3A_1$, black) and ($^3A_2$, red).

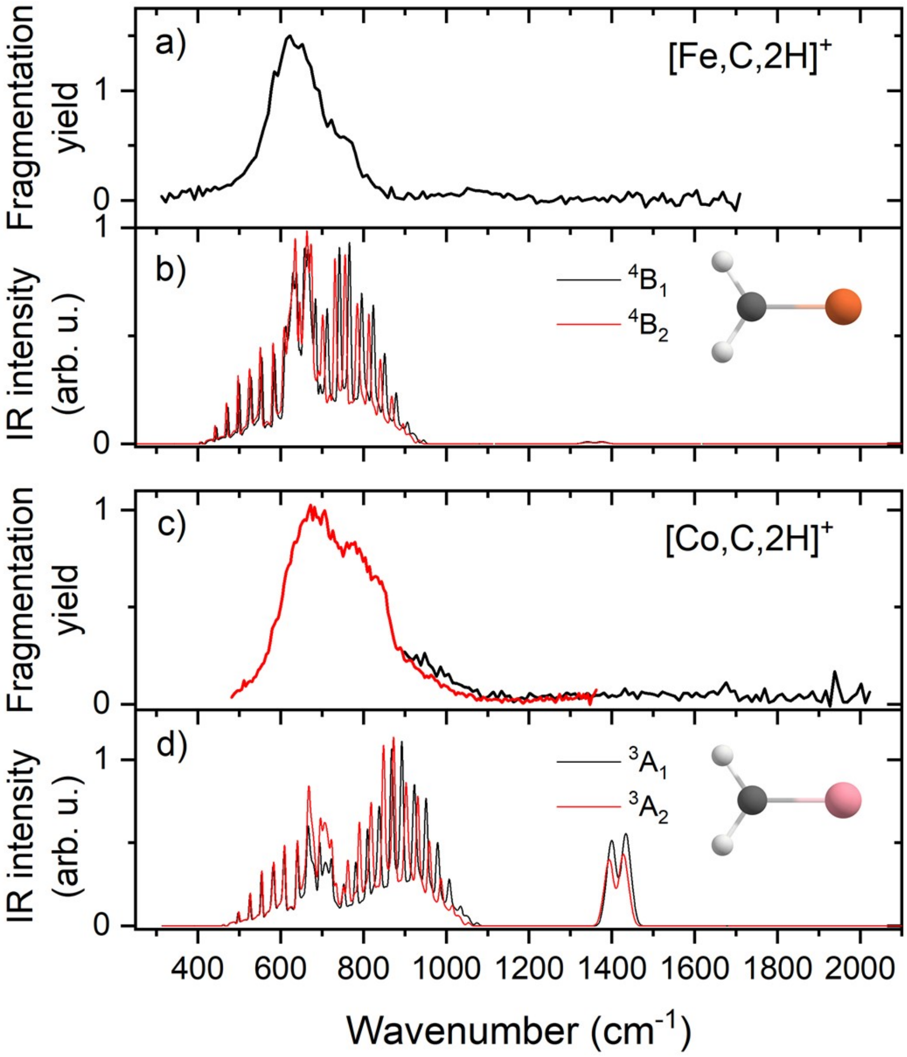

Figure S5: Experimental IRMPD spectra of $[Fe,C,2H]^+$ (panel a) and $[Co,C,2H]^+$ (panel c). Comparison of the spectra calculated at the CCSD/def2-TZVPPD level with rovibrational simulations for (panel b) $FeCH_2^+$ ($^4B_1$, black) and ($^4B_2$, red) and (panel d) $CoCH_2^+$ ($^3A_1$, black) and ($^3A_2$, red).

Table S1: Ground and excited electronic states for $[M,C,2H]^+$ isomers with electronic occupation for selected states calculated at the B3LYP/def2-TZVPPD level. The 0 K relative energy E_{Rel} is given with respect to the lowest energy isomer for the metal involved.

Species	State	E_{Rel} (eV)	Occupation
$FeCH_2^+$	$^4B_1$	0.000	$(1a_1)^2(1b_1)^2(2a_1)^2(1a_2)^1(3a_1)^1(1b_2)^1$
	$^4B_2$	0.013	$(1a_1)^2(1b_1)^2(2a_1)^1(1a_2)^2(3a_1)^1(1b_2)^1$
	$^4A_2$	0.179	$(1a_1)^2(1b_1)^2(2a_1)^1(1a_2)^1(3a_1)^1(1b_2)^2$
	$^4B_1$	0.635	$(1a_1)^2(1b_1)^2(2a_1)^1(1a_2)^1(3a_1)^2(1b_2)^1$
	$^4A_1$	1.009	$(1a_1)^2(1b_1)^2(2a_1)^1(1a_2)^1(3a_1)^1(1b_2)^1(2b_1)^1$
	$^6B_1$	0.826	$(1a_1)^2(1b_1)^1(2a_1)^2(1a_2)^1(3a_1)^1(1b_2)^1(2b_1)^1$
	$^2A''$	1.038	$(1a_1)^2(1b_1)^2(2a_1)^2(1a_2)^1(3a_1)^1(1b_2)^1$
	$^2B_1$	1.052	$(1a_1)^2(1b_1)^2(2a_1)^2(1a_2)^1(3a_1)^1(1b_2)^1$
	$^2A_2$	1.061	$(1a_1)^2(1b_1)^2(2a_1)^2(1a_2)^1(3a_1)^0(1b_2)^2$
	$^2B_1$	1.328	$(1a_1)^2(1b_1)^2(2a_1)^2(1a_2)^1(3a_1)^1(1b_2)^1$
	$^2A_2$	1.419	$(1a_1)^2(1b_1)^2(2a_1)^2(1a_2)^1(3a_1)^0(1b_2)^2$
	$^2A_1$	2.008	
	$^2A_1$	2.367	
$HFeCH^+$	$^6A''$	3.323	
$(H_2)FeC^+$	$^2A'$	2.695	
	$^4A''$	3.595	
	$^6A'$	4.371	
$HHFeC^+$	$^4A''$	5.544	
$CoCH_2^+$	$^3A_2$	0.000	$(1a_1)^2(1b_1)^2(2a_1)^2(1a_2)^1(3a_1)^1(1b_2)^2$
	$^3A_1$	0.050	$(1a_1)^2(1b_1)^2(2a_1)^1(1a_2)^2(3a_1)^1(1b_2)^2$
	$^3B_1$	0.412	$(1a_1)^2(1b_1)^2(2a_1)^2(1a_2)^1(3a_1)^2(1b_2)^1$
	$^3B_2$	0.898	$(1a_1)^2(1b_1)^2(2a_1)^2(1a_2)^2(3a_1)^1(1b_2)^1$
	$^3A_1$	1.068	$(1a_1)^2(1b_1)^2(2a_1)^2(1a_2)^1(3a_1)^1(1b_2)^1(2b_1)^1$
	$^3A_1$	1.906	
	$^1A_1$	0.714	$(1a_1)^2(1b_1)^2(2a_1)^1(1a_2)^2(3a_1)^1(1b_2)^2$
	$^1A_2$	0.771	$(1a_1)^2(1b_1)^2(2a_1)^2(1a_2)^1(3a_1)^1(1b_2)^2$
	$^1A_1$	0.851	$(1a_1)^2(1b_1)^2(2a_1)^1(1a_2)^2(3a_1)^1(1b_2)^2$
	$^1A_1$	1.711	$(1a_1)^2(1b_1)^2(2a_1)^2(1a_2)^2(3a_1)^0(1b_2)^2$
	$^1A_1$	2.877	
	$^1A_1$	3.005	
	$^5A'$	0.860	
	$^5A''$	0.943	
	$^5B_2$	1.409	
$HCoCH^+$	$^5A'$	3.258	
	$^1A'$	3.670	
$(H_2)CoC^+$	$^1A'$	3.086	
	$^3A'$	3.414	
	$^5B_1$	4.220	

Table S2. Intensities of vibrational modes relative to those for the CH_2 wag (in %) calculated at the B3LYP/CCSD level. Notable differences are bolded.

MCH_2^+	State	Intensity (mode)/Intensity (CH_2 wag) x 100		
		CH_2 rock	M-C stretch	CH_2 scissors
$FeCH_2^+$	$^4B_1$	35/42	**5/41**	1/1
$RuCH_2^+$	$^4B_1$	4/12	0/0.1	0/1
$OsCH_2^+$	$^4B_1$	5/18	0.4/0.3	1/0.1
$CoCH_2^+$	$^3A_2$	41/41	**0.05/25**	**3/23**
$RhCH_2^+$ [a]	$^1A_1$	0/1	24/30	8/22
$RhCH_2^+$ [a]	$^3A_2$	9/6	23/23	6/2
$IrCH_2^+$	$^3A_2$	15/15	35/24	13/4

[a] CCSD values are from EOM-SF_CCSD calculations provided in Wensink, F. J.; Smink, C. E.; Steele, R. P.; Armentrout, P. B.; Bakker, J. M. *Phys. Chem. Chem. Phys.*, submitted for publication.
